# A Retrospective Comparative Study Assessing Patients With Acute Appendicitis During the Pre and Through Lebanese Financial Crisis

**DOI:** 10.7759/cureus.28518

**Published:** 2022-08-29

**Authors:** Nagham Bazzi, Samer Dbouk, Sadek Jaber, Ali Msheik, Mhd Firas Safadi, Zaynab Shaalan, Mariam Bazzi

**Affiliations:** 1 General Surgery, Lebanese University, Beirut, LBN; 2 General Surgery, Al Zahraa Hospital University Medical Center, Beirut, LBN; 3 Orthopaedics and Traumatology, Lebanese University, Beirut, LBN; 4 Neurological Surgery, Lebanese University, Beirut, LBN; 5 General Surgery, University Hospital Carl Gustav Carus, Dresden, DEU; 6 Faculty of Health Sciences, Beirut Arab University, Beirut, LBN; 7 Faculty of Pharmacy, Saint Joseph University, Beirut, LBN

**Keywords:** s: appendectomy, lebanese crisis, financial crisis, complicated appendicitis, s: acute appendicitis

## Abstract

Introduction

In the past three years, Lebanon, a country located in the Middle East, has faced a severe financial crisis. This crisis had many effects on several sectors in Lebanon, including the healthcare sector. The authors expected an increase in the rate of complicated appendicitis after the crisis due to the shortage of medical supplies. The aim of the study was to compare the rate of complicated acute appendicitis before and after the Lebanese crisis.

Methods

The study included two groups of patients with acute appendicitis. The first group included patients admitted in the period between November 2018 and November 2019 (before the crisis). The second group included patients admitted between November 2020 and November 2021 (during the crisis). The data were collected retrospectively and analyzed using SPSS software (version 25.00) (SPSS Inc., Chicago, IL). Ethical approval was obtained and the study was registered at Al Zahraa hospital, University Medical Center in Beirut, Lebanon.

Results

The study included 49 patients in the first group (before the crisis) and 46 patients in the second group (after the crisis). The percentage of complicated appendicitis has increased from 22.4% before the crisis to 28.3% during the crisis. The study showed a statistically significant difference between the two groups in the white blood cell count (10,831 versus 7180 cu.mm, respectively, p=0.006), the operating time (59.9 versus 79.0 minutes, respectively; p=0.004), the need to obtain an intra-peritoneal swab for bacterial culture (83.7% versus 58.7%, respectively; p=0.007), and the need for intra-abdominal abscess drainage (6.1% versus 28.3%, respectively; p=0.004). There were no significant differences in the demographics, the duration of postoperative antibiotic use, the duration of stay in the hospital, and the postoperative complications in the first month following surgery.

Conclusion

Due to the decreased financial income, the high cost of medical care during the Lebanese crisis, and the delay of patients’ presentation to the hospital, the rate of complicated appendicitis increased during the crisis.

## Introduction

For the two years beginning October 2019, Lebanon has been mired in economic and political challenges, exposing its population to severe health repercussions [1,2]. In October 2019, Lebanese people took to the streets calling for accountability and change [3]. The incidents beginning in October 2019 caused a political imbalance [4,5], and the COVID-19 pandemic further compounded the situation, leading to a devastating decrease in the quality of life [4,5]. The aftermath of the August 4, 2020 blast intensified the Lebanese debacle and exacerbated the negative impact on the economy and health system [2].

The healthcare system in Lebanon is crumbling amidst the political crisis and is among the most affected sectors [4,6,7]. Due to the financial crisis and the dwindling medical supplies, many Lebanese are not able to afford appropriate medical care. Médecins sans frontières has reported that "People could die now from totally avoidable and otherwise easily treatable causes, just because hospitals do not have electricity, the right supplies, or staff" [8]. Though a study reported a decline in the death rate for appendicitis, studies documenting the extent of evitable causes of appendicitis deaths in low-income countries are scant [9]. The main reason for the complicated course is the delay in seeking medical attention, which in our case can be attributed to the financial crisis and inadequate hospital resources [10].

In this retrospective study, we compared the results of acute appendicitis in two groups of patients before and during the crisis. The study aims to assess the variation in acute appendicitis presentation (acute versus complicated) before and during the crisis.

## Materials and methods

Study design and ethical approval

A retrospective comparative study including two groups of patients with acute appendicitis was conducted. The first group included patients admitted with the diagnosis of acute appendicitis in the period between November 2018 and November 2019 (before the crisis). The second group included patients admitted between November 2020 and November 2021 (during the crisis). All the patients were treated at Al Zahraa University Medical Center in Beirut, Lebanon. Ethical approval was obtained from the Institutional Review Board (IRB) at Al Zahraa Hospital University Medical Center, Beirut, Lebanon (Approval Number: 1/2022). The study was conducted after obtaining IRB approval. Standard protocols regarding anonymity and confidentiality were respected.

Inclusion and exclusion criteria

Participants were included based on all of the following criteria: A preoperative diagnosis of acute appendicitis according to clinical manifestations, laboratory tests, and imaging findings. The absence of significant organ dysfunction or other surgical contraindications in the preoperative evaluation; an age range between 18 and 80 years [11,12]. The exclusion criteria include all patients who do not meet the inclusion criteria and pregnant or lactating women.

Data collection and end points

The data were collected retrospectively by reviewing patients’ files. All patients meeting the inclusion criteria were included. The collected data covered general participants’ characteristics such as age, sex, smoking status, income per month, insurance coverage, past medical history, and past surgical history. Information about the course of the disease, including symptoms, examination findings, investigations, antibiotic coverage, and details of the procedure, was collected [11-13]. For this study, we considered any case that required drainage as complicated appendicitis.

The primary endpoints of the study in each arm (before and during the Lebanese crisis) included the rate of complicated appendectomy. The secondary endpoints were the operative time, investigations and tests used, antibiotic coverage, appendectomy timing, operative time, postoperative complications, and length of hospital stay.

Statistical analysis

The generated database was analyzed using the SPSS software (version 25.0, SPSS Inc., Chicago, IL). The used tests were an independent sample t-test for quantitative variables (such as age and duration of antibiotic use) and the Chi-square test for the association between two binomial qualitative variables (such as sex, smoking status, and abscess drainage). Fisher’s exact test was used for variables with incomplete data (such as alcoholic status and the timing of symptoms). Cramer’s V test was applied to qualitative variables with three or more categories. Examples include preoperative imaging modalities, routes of antibiotic administration, and postoperative complications. The significant p-value was set according to Levene’s test, assuming equal variance or unequal variance. The odds ratio (OR) was tested when the p-value was significant to assess the strength of the relationship between two qualitative variables.

## Results

General demographics and characteristics

The study included a total of 49 and 46 patients meeting the inclusion criteria in groups 1 and 2, respectively. The total number of surgeries performed at the hospital in the first and second periods was 7581 and 6354, respectively. The total number of patients who presented with acute appendicitis but did not meet the inclusion criteria was 25 and 14 in the first and second periods, respectively. The rate of complicated appendicitis in group 1 was 22.4% (11 of 49 patients) compared to 28.3% (13 of 46 patients) in group 2 (p-value=0.515). There was also no significant difference between the two groups concerning insurance coverage (Table 1).

**Table 1:** Summary of general demographics and characteristics of the study population

Characteristics	Group 1 (2018-2019, before the crisis)	Group 2 (2020-2021, after the crisis)	P-value^*^
Total number of patients	49	46	-
Simple acute appendicitis	38 (77.6%)	33 (71.7%)	0.515
Complicated acute appendicitis	11 (22.4%)	13 (28.3%)	
Mean age (years)	34.5	35.7	0.673
Male	29 (48.3%)	31 (51.7%)	0.407
Female	20 (40.8%)	15 (42.9%)	
Smoker	27 (55.1%)	18 (39.1%)	0.119
Non-smoker	22 (44.9%)	28 (60.9%)	
Alcoholic	2 (4.1%)	1 (2.2%)	1.000
Non-alcoholic	47 (95.9%)	45 (97.8%)	
Operation funding: covered financially	40 (81.6%)	32 (69.6%)	0.170
Operation funding: uncovered financially	9 (18.4%)	14 (30.4%)	

Pre- and intra-operative characteristics

There were no significant differences in the development of symptoms or the laboratory findings between the two groups. The White Blood Cell (WBC) count was significantly higher in the first group: 10,831, compared with the second group: 7,180 with a p value of 0.006. No significant difference was noted in the other laboratory values including the C-reactive protein (CRP). The routine antibiotic coverage included either multiple antibiotics from the outset or starting with a single antibiotic with adding more medications in complicated cases. Multiple antibiotics (prior and during the admission) were administered in 57.1% of group 1 patients as compared to 82.6% of group 2 patients. Starting with "only one antibiotic" was applied for 42.9% of group 1 patients and to 15.2% in group 2 patients. Cramer’s value of 0.315 indicated a moderate association between the variables. Computed tomography (CT) scan with intravenous (IV) and per Os (PO) contrast was the most common imaging technique used in both groups. Intra-peritoneal culture in ruptured appendicitis was obtained in 16.3% of group 1 patients period compared to 41.3% of group 2 patients. This association was statistically significant with a p-value of 0.007. The average operating time in group 1 patients was 59.9±19.4 minutes compared to 79.0±38.6 minutes in group 2 patients (Table 2).

**Table 2:** Preoperative and intra-operative characteristics of the study population *Emergent, within 3 hours of presentation or as soon as possible; urgent, within 3-8 hours or at the next available time; semi-elective, within 8-12 hours. SD: standard deviation, CRP: C-reactive protein, WBC: white blood cell, CT: computed tomography, IV: intravenous, PO: per os.

Characteristics	Group 1 (2018-2019, before the crisis)	Group 2 (2020-2021, after the crisis)	P/Cramer’s V
Mean time of symptoms (days) ± SD	40.4 ± 59.7	55.1 ± 117.9	p=0.441
Mean highest temperature (°C) ± SD	37.5 ± 1.0	37.6 ± 0.9	p=0.667
Mean CRP (mg/dL) ± SD	116.6 ± 40.2	128.1 ± 55.7	0.332
Mean WBC (cu.mm) ± SD	10,831 ± 5,225	7,180 ± 7,086	0.006
Mean Neutrophils (%) ± SD	69.6 ± 18.1	64.0 ± 25.5	0.227
CT with IV contrast	14 (28.6%)	9 (19.6%)	Cramer’s V=0.185
CT with IV & oral contrast	27 (55.1%)	30 (65.2%)	
CT with oral contrast	1 (2.0%)	1 (2.2%)	
Imaging not reported	7 (14.3%)	6 (13%)	
Antibiotic Coverage Multiple antibiotics	28 (57.1%)	38 (82.6%)	Cramer’s V=0.315
Antibiotic Coverage: Single antibiotic, then multiple if ruptured	21 (42.9%)	8 (17.4%)	
Route of pre-operative antibiotics for complicated appendicitis: IV	43 (97.7%)	45 (100.0%)	p=0.494
Route of pre-operative antibiotics for complicated appendicitis: PO	1 (2.3%)	0 (0%)	
Did the patient received appendectomy: No	1 (2%)	1 (2.2%)	p=1.000
Did the patient receive appendectomy: Yes	48 (98%)	45 (97.8%)	
Timing of appendectomy^*^: Emergent	23 (46.9%)	7 (15.2%)	Cramer’s V=0.004
Timing of appendectomy^*^: Urgent	16 (32.7%)	14 (30.4)	
Timing of appendectomy^*^: Semi-elective	7 (14.3%)	18 (39.1%)	
Timing of appendectomy^*^: > 12 hours	2 (4.1%)	6 (13%)	
Timing of appendectomy^*^: No surgery	1 (2%)	1 (2.2%)	
Intra-peritoneal culture in ruptured appendicitis: not performed	41 (83.7%)	27 (58.7%)	p=0.007
Intra-peritoneal culture in ruptured appendicitis: Yes	8 (16.3%)	19 (41.3%)	
Mean operating time (min) ± SD	59.9 ± 19.4	79.0 ± 38.6	p=0.004

Post-operative characteristics

A total of 6.1% of group 1 patients underwent abscess drainage compared to 28.3% of group 2 patients. This difference was statistically significant with a p-value of 0.004 <0.05 (Table 3).

**Table 3:** Post-operative characteristics of the study population SD: standard deviation, IV: intravenous; PO: per os, N/A: not available

Characteristics		Group 1 (2018-2019, before crisis)	Group 2 (2020-2021, after crisis)	P/Cramer’s V
Mean hospital stays (days) ± SD		2.9 ± 1.5	3.1 ± 1.1	p=0.315
Duration of postoperative antibiotics for complicated appendicitis at hospital	24 hours	20 (40.8%)	15 (32.6%)	Cramer’s V=0.890
	48 hours	20 (40.8%)	22 (47.8%)	
	3 days	6 (12.2%)	7 (15.2%)	
	5 days/7 days	2 (4.1%)	1 (2.2%)	
	No surgery	1 (2%)	1 (2.2%)	
Route of postoperative antibiotics for complicated appendicitis at home	IV	1 (2%)	0 (0%)	Cramer’s V=0.535
	None	2 (4.1%)	1 (2.2%)	
	PO	46 (93.9%)	45 (97.8%)	
Average time for resumption of gastrointestinal transit	1 day	19 (38.8%)	14 (30.4%)	Cramer’s V=0.5
	2 days	23 (46.9%)	29 (63%)	
	3 days	4 (8.2%)	2 (4.3%)	
	4 days	1 (2%)	0 (0%)	
	N/A	2 (4.1%)	1 (2.2%)	
Postoperative complications in the first month	Intra-abdominal abscess	0 (0%)	1 (2.2%)	Cramer’s V=0.337
	None	49 (100%)	44 (95.7%)	
	Fever/discharge	0 (0%)	1 (2.2%)	
Drainage of abscess	No	46 (93.9%)	33 (71.7%)	p=0.004
	Yes	3 (6.1%)	13 (28.3%)	
Appendectomy after antibiotic therapy	No	31 (63.3%)	25 (54.3%)	p=0.377
	Yes	18 (36.7%)	21 (45.7%)	
Timing of appendectomy after antibiotic therapy	After 1 day	14 (28.6%)	18 39.1%	p=0.185
	After 2 days	2 (4.1%)	3 6.5%	
	Same day	2 (4.1%)	0 (0%)	
	N/A	31 (63.3%)	25 (54.3%)	

## Discussion

The findings of this study show the different presentations of acute appendicitis pre and during the Lebanese crisis. The parameters that showed a significant statistical difference between the two groups included the WBC count, the operating time, the need to obtain an intra-peritoneal culture, and the need for intra-abdominal abscess drainage. Additionally, emergent appendectomies have increased in the second group compared to the first one.

Leukocytosis presents a significant indicator of the severity of appendicitis and the presence of complications [14]. Increased WBC count is more significant in complicated appendicitis than in non-complicated [15]. In our study, the WBC count was lower in the patients who presented during the crisis, although the complication rate was higher in this group. The interpretation of these findings may not be straightforward.

Today, Lebanon is lurching from crisis to crisis, making the medical sector poorly prepared to meet the challenges and putting pressure on existing resources [16]. The financial status that is gripping Lebanon has forced the Lebanese to cut down on elective procedures [17]. To spare the expensive costs, some patients may have chosen to treat themselves with analgesics and antibiotics.

The mean operative time increased from 59.9 ± 19.4 minutes in group 1 patients to 79.0 ± 38.6 minutes in group 2 patients. The average operation time for appendectomy ranged in the studies from 37 to 59 minutes, which matches our pre-crisis findings [18,19]. In addition to the increased rate of complicated cases, the increased operative time in patients treated after the crisis may have other reasons.

First, the majority of patients with complicated appendicitis presented in the second group, therefore the operating time was longer in the second group [20]. Second, an acute shortage of medical staff during a crisis with under serving of the operation rooms can contribute to longer operation times [21]. This shortage can be attributed to multiple factors. Many healthcare providers are underpaid due to the devaluation of the Lebanese pound of more than 80% [17,22]. 

Antibiotic coverage has differed between the two groups. More patients (about 82.6%) received coverage with multiple antibiotics during the crisis than those before it (57.1%). The majority of acute appendicitis cases (70-80%) reported in the literature were not complicated. This explains the common use of single antibiotic coverage instead of multiple antibiotics [23-25]. According to our study, multiple antibiotic usage has increased in the second group. This might be explained by the increase in complicated cases during the crisis. Most patients presented after a deterioration of their condition and only when specialist medical care became inevitable, which explains the need for more antibiotic therapy [17].

Obtaining peritoneal swabs for bacterial culture in patients with appendicitis helps in identifying the potential culprit organisms, especially in cases of perforated appendix and peritonitis [26]. The need for taking peritoneal swabs increased from 16.3% in patients treated before the crisis to 41.3% during the crisis. Again, this reflects the higher rate of complicated cases encountered during the crisis. The COVID-19 lockdown restrictions may have altered the patterns of organisms, simultaneously representing a threat to the Lebanese health sector that was already plagued by medical equipment shortages [27].

Last but not least, the number of patients undergoing abscess drainage during the crisis was higher compared to the first group. Neglected appendicitis can lead to various complications, including perforation, pelvic abscess, peritonitis, portal vein thrombosis, liver abscess, and stool fistula [26]. Due to the high medical costs, many patients present only after the occurrence of complications [17,27]. The need for intra-abdominal abscess drainage in about a quarter of the patients (28.3%) indicates a real problem.

This study may have some drawbacks. First of all, the small sample size of the two groups may not yield precise statistical results. Second, practice differences are present between regions, cities, hospitals, and practitioners, so a study performed in one hospital may not reflect the situation in the whole country. Third, some adapted practices may not comply with the recognized standards of care. Examples may include the routine use of computed tomography in diagnosis or the use of antibiotics, which makes the comparison of the results with the evidence-based literature difficult.

## Conclusions

There has been a significant increase in the incidence of complicated appendicitis during the Lebanese crisis that started in 2019. The crisis affected the economy, supply chains, income levels, and the performance of healthcare facilities. According to our study, the course of common diseases such as acute appendicitis was affected by the delayed presentation of patients. The operating time, emergent appendectomies, and rate of intra-abdominal abscesses, with the need for additional therapeutic interventions, have increased in the second group. The healthcare sector is one of the main affected domains in the national and international crisis, which exaggerates the suffering of people and affects them in the long term. Further studies are now warranted to assess the impact of the Lebanese debacle on further healthcare aspects.
